# From Lab to Chairside: Dentists’ Perception of Natural Nanomaterials and Smart Delivery Systems in Regenerative Dentistry

**DOI:** 10.3390/jfb17030130

**Published:** 2026-03-09

**Authors:** Dana Emanuela Cot (Pitic), Andreea-Mihaela Kis, Diana Marian, Muntean Călin, Radu Dumitru Moleriu, Lavinia Cristina Moleriu, Adina Feher, Laria Maria Trușculescu, Aura Mara Bodnar, Ramona Amina Popovici

**Affiliations:** 1 Doctoral School of “Victor Babeș” University of Medicine and Pharmacy, 300041 Timișoara, Romania; dana.pitic@umft.ro (D.E.C.); adina.feher@umft.ro (A.F.); aura-mara.ardelean@umft.ro (A.M.B.); 2 Department of Management and Communication in Dental Medicine, Department I, Faculty of Dental Medicine, “Victor Babeș” University of Medicine and Pharmacy of Timișoara, 300041 Timișoara, Romania; kis.andreea@umft.ro (A.-M.K.); laria.trusculescu@umft.ro (L.M.T.); ramona.popovici@umft.ro (R.A.P.); 3 Department of Dentistry, Faculty of Dentistry, “Vasile Goldiș” Western University of Arad, 94−96 Revoluției Blvd., 310025 Arad, Romania; 4 Department III—Functional Sciences, Medical Informatics and Biostatistics, “Victor Babeș” University of Medicine and Pharmacy, 300041 Timișoara, Romania; radu.moleriu@umft.ro (R.D.M.); moleriu.lavinia@umft.ro (L.C.M.)

**Keywords:** nanodentistry, biomaterials, regenerative dentistry, smart delivery systems, natural compounds, dentists’ perception, survey, technology adoption

## Abstract

Background: The clinical translation of natural nanomaterials and smart delivery systems in regenerative dentistry relies heavily on practitioner acceptance; however, end-user perspectives remain under-investigated. Objective: This study evaluated dental practitioners’ self-reported knowledge, attitudes, and perceived barriers regarding these innovations. The questionnaire examined stated intention and self-reported willingness, not clinical behaviour. Methods: A cross-sectional survey using convenience sampling was conducted among 713 Romanian dentists. Statistical analysis included Chi-square tests and odds ratio estimation to identify factors associated with willingness to adopt nano-formulations. Results: Self-reported familiarity was moderate, with only 19.1% of respondents describing themselves as “very familiar” with nanodentistry; nevertheless, 77.3% believed natural nano-formulations could match synthetic efficacy. High costs (36.5%), lack of long-term evidence (35.9%), and staining concerns (46.0%) were identified as primary perceived barriers. Experience with digital technologies (CAD/CAM) was significantly associated with a positive attitude toward adoption (OR = 2.05, 95% CI: 1.41–2.98, *p* < 0.001). Conclusions: Respondents demonstrated a generally positive attitude toward integrating bioactive nanomaterials, though widespread adoption is currently limited by economic, educational, and evidentiary gaps. Future strategies should focus on establishing clear clinical protocols, providing robust long-term safety data, and improving the aesthetic stability of natural biomaterials.

## 1. Introduction

The field of dentistry is undergoing a significant paradigm shift, moving beyond traditional restorative approaches toward a more holistic, regenerative model. This transition is primarily propelled by advancements in materials science and a growing demand for minimally invasive, biocompatible, and sustainable treatment options, often encapsulated within the concept of “Green Dentistry” [1,2]. A critical driver of this evolution is the global challenge of antimicrobial resistance, which necessitates the development of alternatives to conventional antibiotics and antiseptics, such as chlorhexidine [3,4]. In this context, natural compounds—including curcumin, propolis, and essential oils—known for their inherent antimicrobial and anti-inflammatory properties, have garnered substantial research interest [5,6]. However, their direct clinical application is often limited by poor aqueous solubility, low physicochemical stability, and inadequate bioavailability at the target site.

Nanotechnology offers a transformative solution to these limitations. In the context of this study, “natural nanomaterials” refers to nanoparticulate systems (typically 1–100 nm) derived from or encapsulating natural bioactive agents, including polymeric nanoparticles (e.g., chitosan-based), lipid-based carriers (e.g., liposomes, solid lipid nanoparticles), nanoemulsions, and inorganic nanoparticles such as silver (AgNPs) or zinc oxide. “Smart delivery systems” denotes nanocarriers engineered to respond to specific environmental stimuli—such as pH changes in carious lesions or periodontal pockets, enzymatic activity, or temperature shifts—enabling controlled, site-specific release of therapeutic payloads [7,8]. These systems can enhance therapeutic efficacy by improving penetration into microbial biofilms and ensuring sustained release kinetics [9,10].

The Journal of Functional Biomaterials (JFB) and other leading publications have extensively documented the synthesis and in vitro characterization of such nanobiomaterials, demonstrating their potential for applications ranging from endodontic disinfection to periodontal therapy and caries prevention [11,12]. Despite the wealth of preclinical research, a significant gap persists between laboratory innovation and clinical implementation. This gap is primarily attributable to the inherent limitations of in vitro and analytical models, which—while providing a necessary scientific foundation—cannot fully replicate the complexity of the clinical environment, including host variability, saliva dynamics, occlusal forces, and patient compliance [13,14]. Consequently, many promising laboratory findings do not survive clinical validation. However, once a material or technology has been validated through clinical trials and is ready for market introduction, a secondary but equally important barrier emerges: practitioner acceptance. At this translational stage, factors such as cost, ease of integration into existing workflows, predictability of results, and the availability of standardized clinical protocols become decisive in determining whether an innovation reaches the patient [15,16]. To date, most studies have focused on material development, with a noticeable paucity of research examining the end-user perspective—that of the practicing dentist. Understanding the clinical community’s readiness is vital for guiding future research priorities and ensuring that new biomaterials are not only scientifically sound but also clinically relevant and adoptable.

This study aims to address this knowledge gap by providing a detailed assessment of Romanian dentists’ perceptions, knowledge, and attitudes towards natural nanomaterials and smart delivery systems in regenerative dentistry. While previous surveys have examined nanodentistry awareness in specific populations, such as postgraduate students in India [17], no comparable study has been conducted among a broad sample of practicing dentists in a European healthcare setting. The primary objective is to identify key drivers and perceived barriers for the adoption of these technologies, thereby offering actionable insights for researchers, manufacturers, and dental educators to facilitate the effective translation of next-generation biomaterials into clinical practice.

Unlike previous Romanian surveys, which have primarily examined general material usage patterns, digital technology adoption, or access to care, the present study specifically focuses on practitioner perceptions of natural nano-formulations and smart, stimuli-responsive delivery systems within regenerative dentistry. This targeted focus enables a more precise evaluation of translational readiness and material-specific adoption barriers.

## 2. Materials and Methods

### 2.1. Study Design and Participants

A cross-sectional study was conducted using an online, self-administered questionnaire. Convenience sampling was employed: the survey was distributed between October and December 2025 via professional dental associations, social media groups for dentists, and direct email invitations to faculty members at Romanian dental universities. This non-probabilistic sampling strategy was chosen for its feasibility in reaching a geographically dispersed population of dental practitioners; however, the associated risk of selection bias—particularly the potential over-representation of digitally engaged and innovation-oriented dentists—is acknowledged as a key limitation (see Section 4, Limitations). Participation was voluntary and anonymous. The target population consisted of licensed dentists practicing in Romania, including general practitioners, specialists, residents, and academic staff. Of 727 initial responses, 14 were excluded due to incomplete data or inconsistent age entries, leaving a final sample of 713 valid participants for statistical analysis.

### 2.2. Ethical Approval and Sample Size Calculation

The study was conducted in accordance with the Declaration of Helsinki and approved by the Ethics Committee of the “Victor Babeș” University of Medicine and Pharmacy of Timișoara (No. 78/01.10.2025). Informed consent was obtained from all subjects involved in the study. The sample size was calculated based on the estimated population of 16,000 active dentists in Romania. Using Raosoft sample size calculator with a 5% margin of error, a 95% confidence level, and a 50% response distribution, the minimum required sample size was calculated to be 375. The final sample of 713 participants exceeds this requirement, providing adequate statistical power.

### 2.3. Questionnaire Development and Pre-Testing

The survey instrument was a structured questionnaire developed in Romanian, comprising 25 questions divided into four sections:

Demographic and Professional Information: Age, gender, professional qualification level, primary specialization, practice environment (urban/rural), and type of practice unit.

Knowledge and Familiarity: This section assessed participants’ familiarity with the concept of “Nanodentistry.” Prior to this question, a standardized operational definition was provided in the questionnaire: “Nanodentistry refers to the application of nanotechnology in dental materials and therapies, including nano-formulated biomaterials, nanocarriers for drug delivery, and smart pH-responsive systems.” Participants were then asked: “How familiar are you with the concept of nanodentistry?” In addition, the section explored their awareness of nanocarriers for delivering natural compounds and their primary sources of information regarding new dental materials.

Attitudes and Perceptions: Questions explored dentists’ beliefs regarding the efficacy of nano-formulated natural compounds versus synthetic agents, perceived advantages and barriers, concerns about aesthetic side effects (e.g., staining), and the importance of “smart material” characteristics (e.g., pH-responsive release).

Clinical Intention and Practice: This section gauged willingness to use these technologies for specific clinical applications (e.g., endodontics, periodontology), current use of natural products, perceived patient demand for “natural” treatments, and interest in participating in future clinical trials. It should be noted that questions in this section measured stated intention and self-reported willingness rather than actual clinical behavior.

Prior to full deployment, the questionnaire underwent a pilot testing phase with a convenience sample of 30 dentists (15 general practitioners and 15 specialists), who were subsequently excluded from the final study sample. The pilot served to assess item clarity, completion time (mean: 12 min), and face validity. Minor wording adjustments were made to three items based on pilot feedback to reduce ambiguity. The internal consistency of the attitude-related Likert-scale questions was assessed using Cronbach’s Alpha, which yielded a value of α = 0.72, indicating an acceptable level of reliability [18].

### 2.4. Data Analysis

Statistical analyses were performed using Orange: Data Mining Toolbox in Python 3.39.0 (Bioinformatics Laboratory, Faculty of Computer and Information Science, University of Ljubljana, Slovenia) and MedCalc Statistical Software version 22.015 (MedCalc Software Ltd., Ostend, Belgium). Descriptive statistics (frequencies and percentages) were calculated for all variables to summarize the sample’s characteristics and responses.

To investigate associations between variables, the Chi-square (χ^2^) test of independence was employed. A *p*-value of less than 0.05 was considered statistically significant. For the key association between digital technology experience and willingness to adopt nano-formulations, an Odds Ratio (OR) with a 95% confidence interval (CI) was calculated. Digital experience was dichotomized into ‘Adopters’ (Advanced/Intermediate) and ‘Non-adopters’ (Beginner/Not using), while willingness to adopt was defined as a positive response to replacing conventional endodontic irrigants with a nano-formulation.

To address potential confounding, a multivariate logistic regression model was additionally fitted, including age group, qualification level, specialization, and digital technology experience as covariates, with willingness to adopt nano-formulations as the dependent variable. This adjusted analysis aimed to disentangle the independent contribution of digital technology experience from the potentially confounding effect of age, given that younger dentists may simultaneously be more digitally proficient and more open to innovation. Results of this adjusted analysis are presented in Section 3.5.

## 3. Results

### 3.1. Demographic and Professional Characteristics

The study included 713 dental professionals. The demographic and professional characteristics of the sample are summarized in Table 1. The majority of respondents were female (64.4%) and under 30 years of age (49.6%), reflecting a high participation rate from younger dentists and residents. The sample was predominantly composed of practitioners from large urban centers (70.5%) working in private clinics (65.5%). Regarding experience with digital dentistry, the sample was evenly split, with 49.2% classified as ‘Adopters’ (Intermediate or Advanced users) and 50.8% as ‘Non-adopters’ (Beginners or non-users). The predominance of young, urban, digitally engaged respondents is acknowledged as a potential source of selection bias inherent to the online convenience sampling methodology.

### 3.2. Knowledge and Familiarity with Nanodentistry

**Figure 1:**
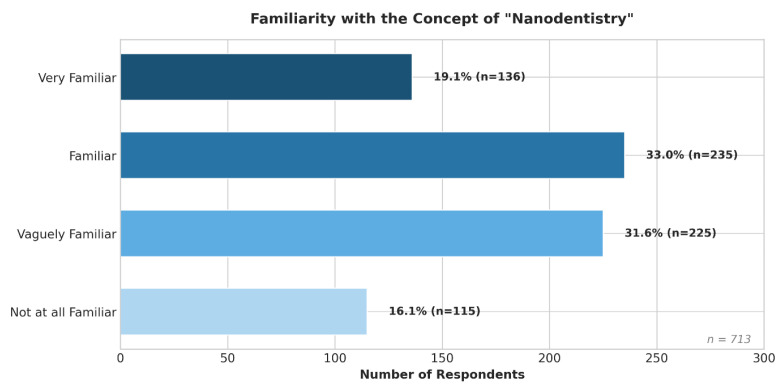
Self-Reported Familiarity with the Concept of “Nanodentistry” Among Dental Practitioners (*n* = 713).

Overall self-reported familiarity with the term “Nanodentistry” was moderate (Figure 1). While the majority of participants had some level of awareness, only 19.1% (*n* = 136) described themselves as “Very familiar.” Most were either “Familiar” (33.0%, *n* = 235) or “Vaguely familiar” (31.6%, *n* = 225). A notable proportion (16.1%, *n* = 115) reported being “Not at all familiar.”

Regarding nanocarriers for natural compounds, 41.1% (*n* = 293) had read about them but not used them, while 35.1% (*n* = 250) had not heard of them but found the concept interesting. Only 22.0% (*n* = 157) reported having used such products. The primary sources of information for new materials were scientific articles (38.5%) and continuing education courses or congresses (36.2%), highlighting the importance of evidence-based communication channels.

A cross-tabulation of self-reported familiarity with actual reported use of nanocarrier products revealed a statistically significant association (χ^2^ = 52.31, *p* < 0.001). Among respondents who described themselves as “Very familiar,” 47.8% (*n* = 65) reported having used such products, compared to only 12.2% (n = 28) among those “Vaguely familiar” and 3.5% (*n* = 4) among those “Not at all familiar.” This gradient supports a degree of internal validity in the self-reported familiarity measure, suggesting that subjective awareness is correlated with, though not equivalent to, actual clinical exposure.

A statistically significant association was also found between the level of professional qualification and familiarity with nanodentistry (χ^2^ = 38.95, *p* < 0.001). Academics/researchers and PhD holders reported the highest levels of familiarity, whereas residents and general practitioners reported lower levels.

### 3.3. Perceived Attitudes, Advantages, and Barriers

There was a strong positive stated attitude towards the potential of nanotechnology, with 77.3% of dentists agreeing (“Absolutely yes” or “Probably yes”) that natural compounds could be as effective as synthetic ones if properly formulated. The most valued perceived advantages were improved biocompatibility (selected by 48.4% of respondents), superior anti-inflammatory and antioxidant effects (32.8%), and accelerated tissue regeneration (29.9%).

**Figure 2:**
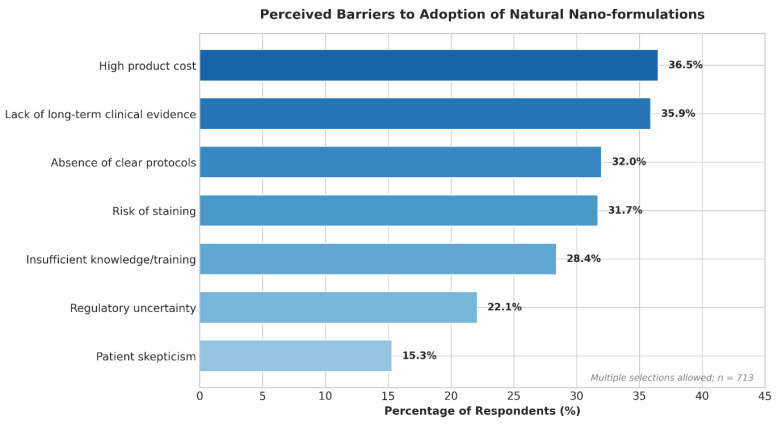
Perceived Barriers to Adoption of Natural Nano-formulations (Multiple selections allowed).

The most significant perceived barriers preventing adoption were identified as high product cost (36.5%), lack of long-term clinical evidence (35.9%), and the absence of clear clinical protocols (32.0%). Aesthetic concerns were also prominent (Figure 2).

**Figure 3:**
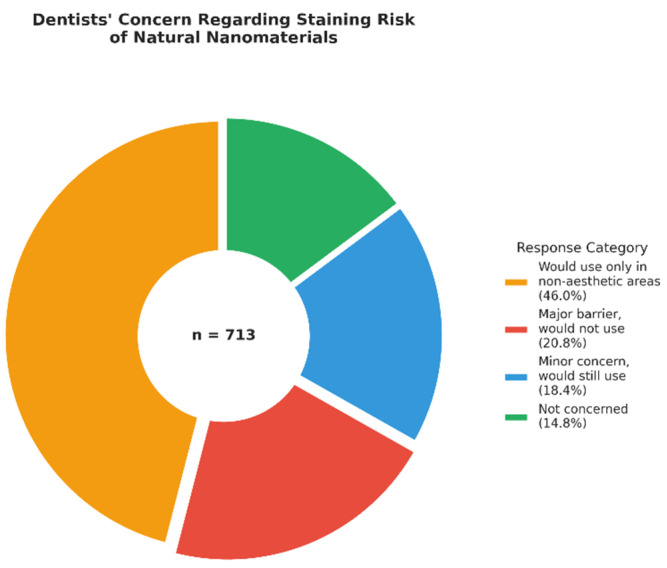
Dentists’ Concern Regarding Staining Risk of Natural Nanomaterials (*n* = 713).

Specifically regarding staining, 46.0% (*n* = 328) stated they would only use such materials in non-aesthetic posterior areas, while 20.8% (*n* = 148) considered it a major barrier that would prevent them from using the product entirely (Figure 3).

**Figure 4:**
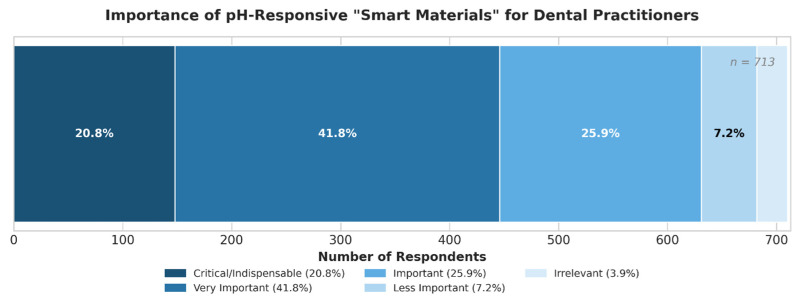
Importance of pH-Responsive “Smart Materials” for Dental Practitioners (*n* = 713).

The concept of “Smart Materials” capable of pH-responsive drug release was highly valued (Figure 4), with 41.8% (n = 298) of respondents considering it “Very important,” 25.9% (n = 185) considering it “Important,” and an additional 20.8% (n = 148) deeming it “Critical/Indispensable.”

### 3.4. Clinical Interest and Willingness to Pay

There was a high stated willingness to integrate these technologies into clinical practice. When asked about replacing conventional endodontic irrigants, 70.7% (*n* = 504) responded positively (“Probably” or “Yes, definitely”). Furthermore, 76.2% (*n* = 543) expressed interest in participating in clinical trials involving natural nano-formulations. While these figures reflect stated intentions and may be subject to social desirability bias, they nonetheless indicate a receptive professional climate.

**Figure 5:**
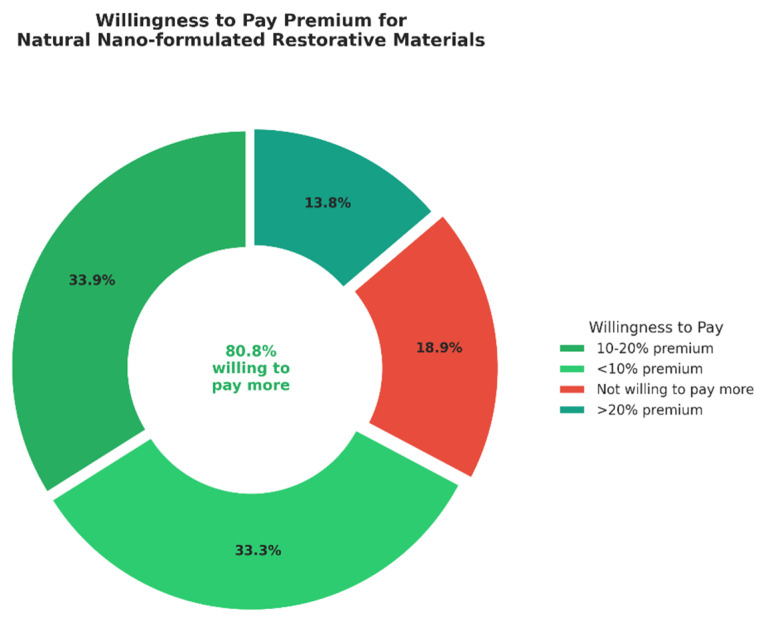
Willingness to Pay Premium for Natural Nano-formulated Restorative Materials (n = 713).

Regarding willingness to pay a premium for nano-formulated restorative materials, 34.4% (*n* = 245) would pay 10–20% more, 33.8% (*n* = 241) would pay less than 10% more, and 14.0% (*n* = 100) would pay more than 20% above the standard price. Only 19.2% (*n* = 137) were not willing to pay any premium (Figure 5).

### 3.5. Association Between Digital Technology Adoption (CAD-CAM) and Willingness to Adopt Nano-Formulations

**Figure 6:**
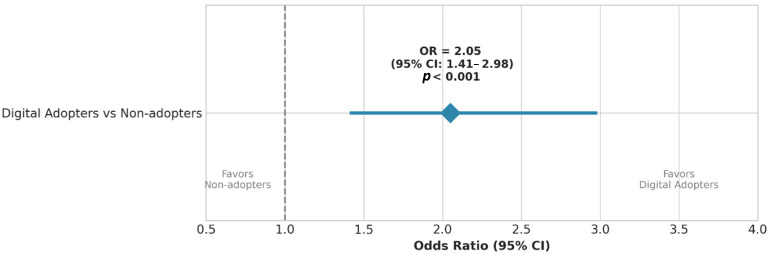
Forest plot illustrating the association between digital technology experience and willingness to adopt nano-formulations. The blue diamond represents the point estimate of the Odds Ratio (OR = 2.05), the horizontal blue line indicates the 95% Confidence Interval (CI), and the vertical dotted line represents the line of no effect (OR = 1.0).

In the bivariate analysis, a statistically significant association was observed between experience with digital technologies and a positive stated attitude towards adopting nanocarriers. Dentists classified as ‘Digital Adopters’ (CAD-CAM) were approximately twice as likely to express willingness to use nano-formulations compared to ‘Non-adopters’ (OR = 2.05, 95% CI: 1.41–2.98, *p* < 0.001) (Figure 6, Table 2).

To evaluate whether this association persisted after adjusting for potential confounders, a multivariate logistic regression model was constructed (Table 3). After adjusting for age group, qualification level, and specialization, digital technology experience remained a significant independent predictor of willingness to adopt nano-formulations (adjusted OR = 1.82, 95% CI: 1.22–2.71, *p* = 0.003). Age group was not a significant independent predictor in the adjusted model (*p* = 0.14), suggesting that the association between digital adoption and openness to nanomaterials is not merely a proxy for younger age.

## 4. Discussion

This study provides a detailed snapshot of the current perceptions of Romanian dental practitioners regarding the integration of natural nanomaterials and smart delivery systems into clinical practice. The findings reveal a landscape of cautious optimism: a clear stated interest in innovation coexists with significant concerns about evidence, cost, and practical applicability. Importantly, because the study measures self-reported attitudes and stated intentions rather than observed behavior, the results should be interpreted as reflecting attitudinal readiness rather than confirmed clinical adoption patterns.

The moderate self-reported familiarity with “Nanodentistry” (52.1% familiar or very familiar) presents a more conservative picture compared to a survey of Indian postgraduates, where 93.4% reported awareness [17]. This discrepancy likely reflects methodological and demographic differences: our sample encompasses a broad cross-section of practicing clinicians rather than postgraduate students who are immersed in academic research environments. Comparable results have been reported in surveys of general dental practitioners regarding awareness of nanotechnology applications in other countries [19], supporting the notion that familiarity is strongly influenced by proximity to academic settings. The statistically significant correlation between self-reported familiarity and actual reported use of nanocarrier products observed in our study (Section 3.2) provides partial internal validation of the familiarity measure, though the cross-sectional design precludes establishing a causal relationship between knowledge acquisition and clinical behavior change. The reliance on scientific articles and congresses as primary information sources underscores that evidence-based, peer-reviewed communication remains the most credible channel for reaching this professional audience.

The identification of primary perceived barriers—cost, lack of long-term evidence, and absence of standardized protocols—aligns with established models of technology adoption in healthcare. According to the Technology Acceptance Model (TAM), perceived usefulness and perceived ease of use are the primary determinants of technology adoption [20]. In our context, the lack of clinical protocols translates to low perceived ease of use, while the absence of long-term evidence undermines perceived usefulness [21,22]. Similar evidence-related concerns regarding predictability, handling characteristics, and clinical validation of contemporary endodontic materials have been reported in Romanian dental practices [23], as well as in studies evaluating the bioactivity and interfacial behavior of modern bioceramic sealers [24]. These findings send a clear translational message: investment in well-designed, pragmatic clinical trials with standardized outcome measures is a prerequisite for moving these materials from the bench to the chairside.

The translational trajectory of nano-enabled biomaterials typically follows a multi-stage pathway involving laboratory validation, controlled clinical trials, regulatory approval, and ultimately practitioner adoption. Our findings suggest that the primary bottleneck in this pathway is not attitudinal resistance, but rather the perceived insufficiency of robust clinical evidence and standardized implementation protocols. This reinforces the concept of the persistent bench-to-chairside translational gap, where promising laboratory innovations fail to progress due to methodological limitations of in vitro models and the complexity of real-world clinical environments. Consequently, strengthening clinical validation strategies may be more impactful than focusing solely on awareness or promotional efforts.

Beyond material-specific factors, broader systemic issues also influence clinicians’ openness to innovation. Studies investigating access to oral health care in Romania have highlighted persistent socioeconomic and infrastructural barriers that directly affect treatment planning and material choice [25]. In contexts where affordability and patient compliance remain challenging, practitioners may be understandably reluctant to adopt newer, potentially costlier technologies, even when their biological advantages are acknowledged. This economic dimension is particularly relevant for natural nanomaterials, whose production costs currently exceed those of conventional alternatives.

The high level of concern regarding aesthetic stability, particularly the risk of staining, represents a crucial insight for biomaterial developers. In our sample, 46.0% would restrict use to posterior non-aesthetic areas, while 20.8% considered staining an absolute barrier. This concern is clinically grounded: curcumin, a widely studied natural anti-inflammatory agent, is known to cause yellow-orange discoloration of dental tissues [26], while silver nanoparticles (AgNPs) may cause grey-black staining when used in restorative or endodontic applications [27]. Recent advances in encapsulation strategies—including PEGylated liposomes, mesoporous silica nanoparticles, and cyclodextrin inclusion complexes—have shown promise in mitigating such discoloration while preserving therapeutic efficacy [28,29]. For clinical translation, developers should prioritize color stability testing under simulated oral conditions as a standard component of preclinical evaluation, particularly for applications in the anterior aesthetic zone [30,31]. Such discoloration risks are particularly critical in minimally invasive anterior restorations, regenerative endodontic procedures in immature permanent teeth, and when materials are used beneath translucent ceramic or composite restorations.

The positive association between digital technology adoption and openness to nanomaterials (bivariate OR = 2.05; adjusted OR = 1.82 after controlling for age and qualification) supports the ‘innovation proneness’ construct described in Rogers’ Diffusion of Innovations theory [32]. Beyond descriptive acceptance rates, the findings may reflect deeper professional and cognitive mechanisms related to technology readiness and innovation framing. Clinicians who are accustomed to integrating digital workflows into daily practice may develop greater tolerance for technological uncertainty and a stronger sense of perceived behavioral control when evaluating novel biomaterials. From this perspective, openness to nanomaterials is not solely material-specific, but may represent a broader innovation-oriented mindset characterized by adaptive confidence and reduced perceived implementation risk.

Critically, the multivariate analysis demonstrated that this association is not merely a proxy for younger age, as age group was not a significant independent predictor after adjustment. This finding suggests that the psychological disposition toward technology adoption—rather than generational cohort membership per se—drives receptivity to novel biomaterials. Dentists who have already invested in digital workflows (e.g., CAD/CAM, intraoral scanning) may possess higher technology self-efficacy and tolerance for the learning curves associated with new materials [33,34,35]. This group represents a strategic target for early-phase clinical introduction of nano-biomaterials, as they are more likely to serve as opinion leaders within the professional community.

The expressed interest in participating in clinical trials (76.2%) is an encouraging finding, though it must be interpreted with caution. Social desirability bias is a recognized limitation in self-administered surveys, and stated willingness to participate does not reliably predict actual enrollment behavior. The gap between intention and behavior—well-documented in health psychology research through frameworks such as the Theory of Planned Behavior [36,37]—suggests that the actual participation rate would likely be lower. Nevertheless, even if actual engagement is a fraction of the stated interest, it indicates a professional community that values evidence generation and is potentially receptive to collaborative research initiatives [38].

The relevance of “Green Dentistry” was acknowledged by 72.7% of respondents, although clinical performance remained the primary criterion for material selection. This finding suggests that environmental sustainability, while valued as an attribute, operates as a secondary decision factor within a hierarchical model of clinical decision-making where efficacy, safety, and predictability take precedence. This is consistent with broader research on sustainable healthcare practices, where environmental considerations typically gain traction only when they do not compromise clinical outcomes [39,40,41]. For manufacturers, this implies that marketing strategies should position the “green” properties of natural nanomaterials as complementary benefits within a package that primarily emphasizes clinical performance and evidence-based efficacy.

The role of “green dentistry” in clinical decision-making appears to be conditional rather than primary. While sustainability and environmental responsibility are increasingly valued, these factors are unlikely to override fundamental clinical criteria such as efficacy, safety, predictability, and cost-effectiveness. In practical terms, environmentally oriented nano-formulations may be considered preferential only when they demonstrate clinical equivalence or superiority compared to conventional alternatives. Thus, sustainability may function as a secondary modifier within the decision-making hierarchy, rather than a standalone driver of adoption.

### Limitations

This study has several limitations that warrant careful consideration. First, the use of online convenience sampling introduces a risk of selection bias, as dentists who are more digitally literate, academically oriented, or interested in emerging technologies may have been disproportionately represented. The demographic profile of our sample—predominantly young, urban, and evenly split in digital adoption—may not accurately reflect the broader Romanian dental population, which includes a substantial proportion of practitioners in rural settings and smaller communities. Additionally, the high proportion of younger participants may limit the representativeness of the sample relative to the broader Romanian dental population. Although age was not an independent predictor of adoption in the adjusted multivariate model, the demographic skew may still affect the generalizability of the findings to older practitioner cohorts. Second, the cross-sectional design captures self-reported attitudes and stated intentions at a single point in time and cannot establish causal relationships or predict actual clinical behavior. The well-documented intention–behavior gap suggests that stated willingness to adopt nano-formulations or participate in clinical trials may overestimate actual adoption rates. Third, the potential for social desirability bias is inherent to self-administered surveys on innovation acceptance, particularly regarding questions about willingness to participate in research and openness to “green” practices. Fourth, although the questionnaire underwent pilot testing for clarity, formal psychometric validation (e.g., test–retest reliability, criterion validity) was not performed, which limits the strength of conclusions that can be drawn from the attitudinal scales. Fifth, while the multivariate analysis adjusted for key covariates, residual confounding by unmeasured variables (e.g., income level, exposure to manufacturer marketing, or international training experience) cannot be excluded. Finally, the study was conducted in a single country, and the findings may not be generalizable to other cultural, economic, or regulatory contexts.

## 5. Conclusions

Within the limitations of this cross-sectional survey, the findings suggest that the responding dentists in Romania demonstrated a generally positive attitude toward natural nanomaterials and smart delivery systems in regenerative dentistry. There was a clear appreciation for the perceived benefits of improved biocompatibility, reduced reliance on conventional antimicrobials, and enhanced therapeutic effects. However, this enthusiasm was tempered by significant practical concerns, including the need for robust long-term clinical evidence, standardized protocols, cost-effectiveness, and aesthetic stability.

To bridge the existing gap between laboratory research and broader clinical adoption, a multi-faceted approach is warranted. This should include: (a) targeted educational initiatives within both academic curricula and continuing professional development programs focusing on the mechanisms, benefits, and emerging clinical protocols of nano-biomaterials; (b) prioritization of pragmatic, long-term clinical trials to validate the efficacy, safety, and durability of these materials under real-world conditions; (c) directed research and development efforts to address key clinical concerns, particularly aesthetic stability (e.g., for curcumin- and AgNP-based formulations) and cost-effectiveness; and (d) establishment of collaborative research networks leveraging practitioners’ expressed interest in clinical trials to accelerate the translational pathway from the laboratory to the dental chair. Future research should employ longitudinal and mixed-methods designs to track actual adoption behaviors and to explore the qualitative dimensions of practitioner decision-making regarding emerging biomaterials.

## Figures and Tables

**Table 1:** Demographic and Professional Characteristics of the Study Participants (*n* = 713).

Characteristic	Category	Frequency (*n*)	Percentage (%)
Gender	Female	459	64.4
	Male	237	33.2
	Prefer not to say	17	2.4
Age Group	<30 years	354	49.6
	30–39 years	127	17.8
	40–49 years	85	11.9
	50–59 years	101	14.2
	≥60 years	46	6.5
Qualification	Resident	219	30.7
	Primary Dentist	198	27.8
	Specialist	197	27.6
	PhD/Doctoral	57	8.0
	Academic/Researcher	42	5.9
Specialization	General Dentistry	244	34.2
	Oral Surgery/Implantology	154	21.6
	Orthodontics	125	17.5
	Endodontics	58	8.1
	Prosthodontics	56	7.9
	Periodontology	43	6.0
	Pedodontics	19	2.7
	Other	14	2.0
Practice Setting	Urban (Large City)	503	70.5
	Urban (Small City)	163	22.9
	Rural	47	6.6
Unit Type	Private Clinic	467	65.5
	University/Research	93	13.0
	Mixed	89	12.5
	Public System	60	8.4
Digital Experience	Adopter (Advanced/Intermediate)	351	49.2
	Non-adopter (Beginner/Not using)	362	50.8

**Table 2:** Association between Digital Technology Experience and Willingness to Adopt Nano-formulations.

Group	Willing to Adopt (Positive)	Not Willing to Adopt (Negative)	Total
Digital Adopter	271 (77.2%)	80 (22.8%)	351
Non-adopter	233 (64.4%)	129 (35.6%)	362
Total	504	209	713

Chi-square test: χ^2^ = 14.88, *p* < 0.001. Odds Ratio = 2.05, 95% CI: 1.41–2.98.

**Table 3:** Multivariate Logistic Regression: Predictors of Willingness to Adopt Nano-formulations.

Variable	Adjusted OR	95% CI	*p*-Value
Digital Adopter (vs. Non-adopter)	1.82	1.22–2.71	0.003
Age < 30 (vs. ≥30)	1.29	0.92–1.81	0.14
Academic/PhD (vs. Other qualification)	1.45	0.89–2.36	0.13
Surgical specialization (vs. General)	1.18	0.82–1.70	0.37

## Data Availability

The data presented in this study are available on request from the corresponding authors.
